# Combination Intensity-Modulated Radiotherapy and Sorafenib Improves Outcomes in Hepatocellular Carcinoma with Portal Vein Tumor Thrombosis

**DOI:** 10.1155/2021/9943683

**Published:** 2021-12-03

**Authors:** Munire Abulimiti, Zhenyu Li, Haifeng Wang, Palida Apiziaji, Yisikandaer Abulimiti, Yao Tan

**Affiliations:** ^1^Department of Digestive Internal Medicine, The Affiliated Tumor Hospital of Xinjiang Medical University, Urumqi 830011, Xinjiang Uygur Autonomous Region, China; ^2^Department of Thoracic and Abdominal Radiotherapy, The Affiliated Tumor Hospital of Xinjiang Medical University, Urumqi 830011, Xinjiang Uygur Autonomous Region, China

## Abstract

**Purpose:**

To compare the difference in outcome of hepatocellular carcinoma (HCC) with portal vein thrombosis (PVTT) between intensity-modulated radiotherapy (IMRT) concurrent with sorafenib and IMRT alone.

**Methods:**

A total of 82 patients with PVTT from 2014 to 2019 were included. Of these, 36 received IMRT concurrent with sorafenib treatment (group A), while 46 underwent IMRT alone (group B). The dose of IMRT was 40.0–62.5 Gy/2–2.5 Gy/4–6 w, and patients received orally administered sorafenib 400 mg twice a day in combination with IMRT. Overall survival (OS), progression-free survival (PFS), and median distant metastasis-free survival (DMFS) were evaluated by using LIFETEST procedure of SAS.

**Results:**

The median survival time was 11.0 months in group A versus 9.0 months in group B. The 1- and 2-year OS in group A were 44.9% and 3.8% versus 28.6% and 2.6% in group B (*P*=0.036), respectively. The median PFS was 6.0 months in group A versus 3.0 months in group B. The 1- and 2-year PFS in group A were 20.7% and 6.9% versus 2.7% and 0.0% in group B (*P*=0.012), respectively. The 1- and 2-year DMFS in group A were 38.0% and 7.9% versus 16.7% and 0.0% in group B (*P*=0.019), respectively. Multivariate analysis showed that Child–Pugh classification, AFP response, and overall response were independent risk factors for OS (*P* < 0.05). There were no significant differences in adverse events except fatigue and skin reactions between the two groups.

**Conclusion:**

Compared with IMRT alone, IMRT concurrent with sorafenib can improve the long-term efficacy of HCC patients with PVTT, without increasing adverse reactions. The patients with Child–Pugh A, overall response, and AFP response obtained better OS.

## 1. Introduction

Hepatocellular carcinoma (HCC) is the sixth most common tumor worldwide [1], and the incidence and mortality of HCC in males ranked second in developing countries [2]. HCC patients are prone to developing portal vein tumor thrombosis (PVTT) due to liver anatomical features and HCC biological characteristics. The incidence of PVTT is 44.0% to 62.6%, while the median survival of patients without standard treatment was only 2.7 months because the presence of PVTT is associated with internal and external liver metastasis, liver dysfunction, portal hypertension, ascites, and other related complications [3].

At present, HCC patients with PVTT can be treated by transarterial chemoembolization (TACE), radiofrequency ablation (RFA), radiotherapy, targeted therapy, or immunotherapy. As conventional radiotherapy is prone to radiation-induced hepatocyte injury, its role in the comprehensive treatment of HCC is limited. Moreover, when the dose in target tumor area did not reach the radical therapeutic level, the dose of normal liver tissue has exceeded the tolerance, leading to insufficient radiation dose and ineffective efficacy. With the rapid development of medical technology, accurate local radiotherapy, such as intensity-modulated radiation therapy (IMRT) and stereotactic body radiation therapy (SBRT), displays a better protection for normal liver tissues than conventional radiotherapy. In this case, the local radiotherapy can increase the dose of local tumor to the therapeutic level, even up to 90.0 Gy and the incidence of radiation-induced liver disease (RILD) is less than 5.0% [4]. Sorafenib and lenvatinib are molecular-targeted drugs that are recognized to prolong the survival time of patients with advanced HCC [5, 6]. Several studies suggested that sorafenib combined with other treatments such as TACE or RFA could improve the overall survival of HCC patients with PVTT [7, 8], albeit there is still controversy about radiotherapy combined with targeted therapy. Brade et al. [9] reported that the objective efficacy of sorafenib combined with external radiotherapy was not improved, whereas the adverse reactions were increased. Zhao et al. [10] treated 63 HCC patients with macrovascular invasion (MVI) by using TACE plus IMRT combined with sorafenib and found an excellent safety profile and clinical efficacy. Here, we retrospectively analyzed the clinical data of 82 HCC patients with PVTT and evaluated the benefits, safety, and prognostic factors of IMRT combined with sorafenib.

## 2. Materials and Methods

### 2.1. Patient Selection

The inclusion criteria were as follows: (1) HCC pathologically diagnosed or confirmed by clinical examination and presence of PVTT identified using either CT scans or MRI scans, (2) Child–Pugh classification A or B (6-7 points), (3) an eastern cooperative oncology group (ECOG) performance status ≤2, (4) normal renal function, (5) no previous liver radiation therapy, (6) the tolerance of normal liver tissue to radiotherapy (normal liver volume ≥700 cm^3^), and (7) patients who have completed treatment and clinical data available for analysis. The following exclusion criteria were as applied: (a) Child–Pugh class C disease with poor liver function, (b) ECOG >2, and (c) extrahepatic metastasis.

82 patients enrolled in this study were diagnosed with HCC with PVTT, based on the NCCN Clinical Practice Guidelines in Oncology (Hepatobiliary Cancers). All the patients underwent clinical treatment at our Hospital between January 2014 to December 2019, with or without TACE performed before. Tumor staging was based on the American Joint Committee on Cancer (AJCC) TNM staging system (7th edition). PVTT typing was defined according to the Chinese Cheng's classification [11]. Eighty-two patients of HCC with PVTT were divided into two groups according to whether sorafenib was synchronized or not: patients (*n* = 36) in group A were treated with IMRT combined with sorafenib, patients (*n* = 46) in group B were treated with IMRT only. All patients signed informed consent for radiotherapy before treatment, while patients in group A provided written informed consent for targeted therapy. This study was approved by the Institutional Ethics Committee of The Affiliated Tumor Hospital of Xinjiang Medical University.

### 2.2. Clinical Treatments

#### 2.2.1. IMRT

Patients lay in the supine position with their hands crossed and held over their forehead. They were fixed to the positioning plate using a hydrolyzed plastic body membrane for reducing the position error and liver motion caused by abdominal breathing. After the body surface and the relative position of the body membrane and frame were marked, patients underwent computed tomography (CT) simulation under calm breathing. Patients without contraindications were asked to undergo magnetic resonance imaging (MRI) scans for optimizing the target and normal structures. CT and MRI images were uploaded to the Varian radiotherapy planning system to delineate the treatment target.

The gross tumor volume (GTV) was defined as the tumor volume that was enhanced in the arterial phase combined with the PVTT volume, which was shown as a filling defect in the portal venous phase of the CT or MRI scan. The clinical target volume (CTV) was contoured with a 0.5 cm margin of the primary mass and 0.5 cm margin of the distal end of the venous thrombus. The planning target volume (PTV) was generated by adding 1 to 1.5 cm margin to the CTV in the cranial-caudal axis as well as 0.5 cm to that in the anterior-posterior and lateral axes for uncertainties in treatment delivery. Radiotherapy was carried out in IMRT using 6 MV X-rays with a linear accelerator (Varian 23EX), while the planned total dose to the PTV was 40–62.5 Gy, with 2–2.5 Gy per fraction, at 5 fraction a week. The RT plan was evaluated by dose-volume histogram, and PTV was calculated to cover 95% of the isodose curve. In regard to the liver, while the absolute normal liver volume (ml, total liver minus PTVs) was >700 ml, the mean dose to normal liver was <23 Gy, and/or the percentage normal liver volume receiving more than 30 Gy (V20) was <30%. The maximum allowable point dose to the stomach and intestine was set to no more than 54 Gy, with the volume of organ receiving >45 Gy less than 15%, while the maximum permissible dose of the cord should be less than 45 Gy. The kidney volume receiving a dose of ≥20 Gy (V20) was less than 50% [10].

#### 2.2.2. Sorafenib

Patients in group A were treated with sorafenib 400 mg twice a day before, during, and after IMRT. In the event of a level 3 to 4 drug-related adverse event, dosage reduction to 200 mg or withdrawal was permitted. Sorafenib treatment was continued as long as possible until disease progression, death, occurrence of adverse events that required termination of treatment, failure of patients to tolerate treatment, or liver function deterioration (total bilirubin >3 mg/dl 4 weeks after cessation of treatment).

#### 2.2.3. Other Treatments

Patients with positive HBV-DNA or HCV-RNA received whole-course antiviral therapy to suppress the infected viruses. Based on the liver function and the general condition, symptomatic and supportive treatment, such as protecting the liver, suppressing acid, and repairing mucous membranes, were given during radiotherapy.

### 2.3. Follow-Up, Response, and Toxicity Assessment

After treatment, patients received regular follow-up and evaluation, including ECOG performance status classification, blood routine examination, biochemical test, coagulation test, serum alpha-fetoprotein (AFP), abdominal ultrasound, CT of the chest and abdomen, and MRI of the liver. The follow-up was continued until the death of patients or May 2020. The mean follow-up period was 10 months (6.0–15.0 months).

AFP levels were monitored before, during, and after radiotherapy. AFP level before radiotherapy was used as the baseline. AFP response was defined as a reduction of ≥20% in AFP level from baseline, while no significant change or in AFP level was classified as AFP nonresponse. Overall response was assessed 12 weeks after radiotherapy according to revised Response Evaluation Criteria in Solid Tumors (RECIST) guidelines (version1.1) [12]. Complete response (CR) was defined as the complete disappearance of target lesions and absence of new lesions for at least 4 weeks. A reduction of at least 30% in the sum of the diameters of the target lesions for at least 4 weeks was judged to be partial response (PR). Stable disease (SD) and progression disease (PD) were, respectively, as a decrease of <30% in the longest diameter or no change of the target lesions and at least a 20% increase in the sum of the maximum diameter of the target lesions or the presence of new lesions. Short-term efficacy was evaluated according to overall response of the primary tumor and PVTT remission, and objective response rate (ORR) was defined as the sum of CR and PR rates. Long-term efficacy was evaluated according to the median overall survival (OS), median progression-free survival (PFS), distant metastasis-free survival (DMFS) and median survival time. Data on missing patients and nontumor-induced deaths were defined as deletion. The recurrence patterns were divided into in-field recurrence, outfield recurrence, and distant metastasis. Among them, in-field recurrence, outfield recurrence, and distant metastasis were defined, respectively, as new lesions or increased tumor size within PTV, the presence of new liver lesions outside PTV, and metastasis beyond the liver, to the lungs, bone, or other organs.

Acute and late toxicities due to radiotherapy were assessed according to the Common Toxicity Criteria Adverse Events version 4.0. RILD was divided into two types: classic RILD and nonclassic RLID. Classic RILD was defined as the presence of a large number of ascites and liver enlargement in a short period, with an increased level of alkaline phosphatase (AKP), to more than twice, or that of alanine aminotransferase (ALT) to more than five times of the standard value. Meanwhile, nonclassic RILD was only damaged liver function, with an increased level of AKP to more than twice or that of ALT to more than five times of standard value and the absence of liver enlargement and ascites.

### 2.4. Statistical Analysis

Chi-square test or Fisher exact test was used to compare clinical variables between two groups. Mann–Whitney *U* test was used to compare ordinal data. LIFETEST procedure of SAS 9.4 was used to calculate OS, PFS, and DMFS and present the survival curves. Univariate analysis and the Cox proportional hazards model were performed using SPSS version 26.0. Factors with a *p* value less than 0.1 in univariate analysis were further tested in multivariate analysis, using the Cox proportional hazards model. *P* < 0.05 was considered to be statistically significant.

## 3. Results

### 3.1. Patient Characteristics

Among 82 patients, 67 were males and 15 were females, with a median age of 55 years (49.2–60.0 years). The average maximum diameter of the tumor was 6.5 cm (4.0–9.2 cm). According to AJCC staging criteria, there were 47 in stage III and 35 in stage IV. PVTT was classified as type II in 25 patients, type III in 44 patients, and type IV in 13 patients. Most patients had a relatively good condition with ECOG performance status ranging from 0 to 1. While 66 patients had underlying hepatitis B, six patients were positive for hepatitis C. Child–Pugh classification included Child–Pugh A in 79 patients and Child–Pugh B in 3 patients. There were no statistical significant differences in gender, age, tumor size, AJCC TNM stage, ECOG performance status, ALT levels before treatment, AFP levels before radiotherapy, Child–Pugh classification, PVTT typing, etiology, antiviral therapy, and total radiotherapy dose between 2 groups (*P* > 0.05). Further patient characteristics were summarized in Table 1.

### 3.2. Overall Response and AFP Response

Among 36 patients in group A with the ORR of 61.1%, 22, 10, and 4 achieved PR, SD, and PD, respectively. Meanwhile, out of 46 patients in group B with the ORR of 45.7%, 1, 20, 13, and 12 achieved CR, PR, SD, and PD, respectively. Notably, no statistically significant differences in overall response was detected between the two groups. The results were summarized in Table 2.

AFP response (≥20% reduction) was achieved in 22 (61.1%) patients of group A, and 13 (28.3%) patients of group B. Increase levels of AFP were observed in 3(8.3%) and 6 (13.0%) patients of group A and group B, respectively. Strikingly, a statistically significant difference in the AFP response was found between the two groups (*P*=0.003) (Table 2).

### 3.3. Outcomes

The median follow-up time after treatment was 10 months (6.0–15.0 months); it was 11.0 months (8.0–19.0 months) in group A and 9.0 months (6.0–12.3 months) in group B. Among all the patients, 5 (6.1%) including 2 in group A and 3 in group B were lost to follow-up, while 67 (81.7%) died at the end of the study.

**Figure 1:**
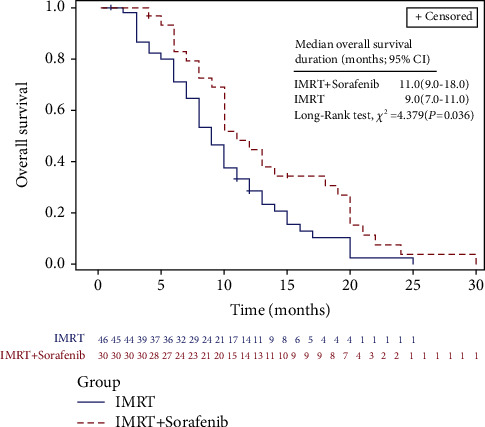
Comparison of LIFETEST of OS between IMRT concurrent with sorafenib (group A) and IMRT alone (group B).

**Figure 2:**
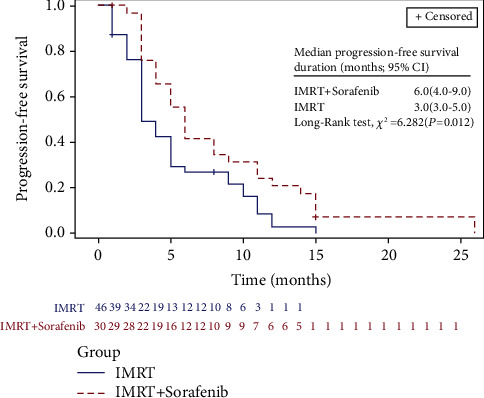
Comparison of LIFETEST of PFS between IMRT concurrent with sorafenib (group A) and IMRT alone (group B).

LIFETEST procedure of SAS showed significant differences in OS and PFS between groups. The median OS was 10.0 months (95% CI, 8.0 to 12.0 months) for this cohort of patients; as depicted in Figure 1, it was 11.0 months (95% CI, 9.0 to 18.0 months) in group A versus 9.0 months (95% CI, 7.0 to 11.0 months) in group B (*P*=0.036). Notably, the 6, 12, 18, and 24 months OS of patients in group A and group B were 82.9%, 44.8%, 30.7%, and 3.8% versus 71.1%, 28.6%, 10.4%, and 2.6%, respectively. In the meantime, median PFS was 5.0 months (95% CI, 3.0 to 6.0 months) among the 82 patients; it was 6.0 months (95% CI, 4.0 to 9.0 months) in group A versus 3.0 months (95% CI, 3.0 to 5.0 months) in group B (*P*=0.012) (Figure 2). The 6, 12, 18, and 24 months PFS of patients in group A and group B were 41.4%, 20.7%, 6.9%, and 6.9% versus 26.8%, 2.7%, 0.0%, and 0.0%, respectively.

**Figure 3:**
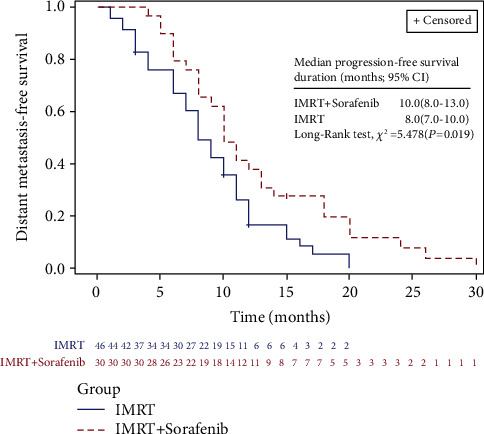
Comparison of LIFETEST of DMFS between IMRT concurrent with sorafenib (group A) and IMRT alone (group B).

The main recurrence pattern in group A was found to be the outfield intrahepatic recurrence, occurring in 14 patients (38.9%), while in-field intrahepatic recurrence developing in 16 patients (34.8%) was identified as the major pattern of recurrence in group B. In addition, distant metastasis (extrahepatic failure) was detected in 6 patients (16.7%) of group A and 8 patients (17.4%) of group B, in which 11, 2, and 1 had lung metastasis, adrenal metastasis, and multiple systemic metastases, respectively. Median DMFS was 10.0 months (95% CI, 8.0 to 11.0 months) for this cohort; it was 10.0 months (95% CI, 7.8 to 13.0 months) in group A versus 8.0 months (95% CI, 7.0 to 10.0 months) in group B (*P*=0.019) (Figure 3). The 6, 12, and 24 months DMFS of patients in group A and group B were 79.4%, 38.0%, and 7.9% versus 67.0%, 16.7%, and 0.0%, respectively. Patients in group A achieved a better DMFS than those in group B, albeit there was no statistically significant difference in the failure pattern between the two groups (*P* > 0.05) (Table 2).

The results of univariate and multivariate analyses were presented in Table 3. Univariate analysis revealed that gender, Child–Pugh classification, pretreatment levels of AFP, AFP response, radiation dose, combination with sorafenib, and overall response were significant prognostic factors for OS. There was a trend toward a better OS for males (11.0 months versus 8.0 months in females), Child–Pugh class A (10.0 months versus 4.0 months in Child–Pugh class B), AFP responders (13.0 months versus 9.0 months in AFP nonresponders), patients receiving higher dose of radiation (13.0 months versus 10.0 months in those receiving lower dose of radiation), patients undergoing IMRT combined with sorafenib (13.0 months versus 9.0 months in those without sorafenib), and overall responders (13.0 months versus 8.0 months in tumor nonresponders). Moreover, multivariate analysis showed that Child–Pugh classification, serum alpha-fetoprotein (AFP) response, and overall response were significantly associated with OS (*P*=0.010, 0.009, and 0.001, respectively).

### 3.4. Toxicity

Treatment-related acute toxicities were summarized in Table 4. An increase in the incidence of grade 1-2 nausea, anorexia, abdominal pain, fatigue, and skin reactions was found in group A (41.7%, 61.1%, 55.6%, 75.0%, and 55.6%, respectively) compared with group B (28.3%, 50.0%, 37.0%, 52.1%, and 34.8%, respectively), albeit there were no significant differences in adverse events except fatigue and skin reactions between the two groups. Among all the patients, two developed grade 2 hand-foot syndrome, and the dose of sorafenib was adjusted to 400 mg per day. The hematologic toxicity mainly included leukopenia and thrombocytopenia. In this case, the grade 3 thrombocytopenia was present in 5 (13.9%) patients of group A, and 6 (13.0%) patients of group B and 5 patients including grade 3 increased level of gamma-glutamyl transpeptidase (GGT). Although grade 3 adverse reactions occurred in both groups, patients were able to continue treatment after symptomatic treatment. Notably, there was no obvious statistical difference in the incidence of hepatotoxicity (*P* > 0.05) between the two groups, albeit a higher rate of hepatotoxicity was observed in group A. Besides, none of the patients developed RILD.

## 4. Discussion

In recent years, there have been growing treatment options available for HCC patients with PVTT. However, the international consensus on the treatment of HCC with PVTT has not been established. Gomaa et al. [13] compared different staging systems for predicting prognosis and survival in HCC patients and concluded that the Barcelona clinic liver cancer (BCLC) staging system provided the best prognostic stratification for HCC patients. Clinical Practice Guidelines issued by the European Association for the Study of the Liver (EASL) classify macrovascular invasion (either segmental or portal invasion) as advanced HCC (BCLC stage C), recommending sorafenib or lenvatinib as the standard first-line systemic therapy [14]. However, there is striking evidence that the corresponding comprehensive treatment can be selected based on liver function, PVTT typing, tumor resectability, and distant metastasis to improve survival rate. Kok et al. [15] classified patients into the sorafenib-TACE group and sorafenib-alone group to compare survival, mortality, and safety between the two groups. Strikingly, they found that the combination of TACE and sorafenib improved survival (381 versus 204 days in sorafenib-alone group), while causing a 26% reduction in mortality. Yoon et al. [16] compared the efficacy and safety between TACE plus external beam radiotherapy (EBRT) and sorafenib for HCC patients with MVI. The comparative study showed that while the TACE plus RT significantly improved overall survival (55.0 versus 43.0 weeks for sorafenib), as well as the median time to progression (31.0 versus 11.7 weeks for sorafenib), 5 patients were treated with curative surgical resection. He et al. [17] investigated the efficacy and safety of sorafenib plus hepatic arterial infusion chemotherapy (HAIC) of oxaliplatin, fluorouracil, and leucovorin (FOLFOX) for HCC with PVTT in comparison with sorafenib. The group of sorafenib plus HAIC of the FOLFOX achieved a higher response rate (40.8% versus 2.46%) and more prolonged median OS (13.37 versus 7.13 months) and PFS (7.03 versus 2.6 months) than sorafenib group. These lines of evidence suggest that the combination therapy could be superior to sorafenib monotherapy.

AFP has been extensively investigated as a useful screening, diagnosis, surveillance, recurrence monitoring, and prognostic prediction in HCC [18]. Chou et al. [19] analyzed the change in AFP levels among 81 HCC patients receiving oxaliplatin-based chemotherapy. In the study, they found that the AFP decline group achieved longer median OS and PFS than the AFP nondecline group (12.3 versus 3.0 months and 7.0 versus 2.3 months, respectively) and the unit change in AFP level had a predictive value for PFS and OS. Sánchez et al. [20] classified 167 patients treated with sorafenib into two groups: one with AFP reduction ≤20% (nonresponders) and the other with AFP reduction >20% (responders). They observed that the median OS was significantly decreased in those patients with baseline AFP levels >200 ng/ml compared with those with AFP levels ≤200 ng/ml (8 versus 14 months). Moreover, patients in the responder group exhibited a significantly longer median OS (18.0 months) than those in the nonresponder group (10.0 months) (*P*=0.004), indicating that a >20% drop in AFP 6–8 weeks may serve as a useful factor for prediction of response to sorafenib. In the present study, we showed that group A with a median OS of 14 months had more patients achieving a >20% reduction of AFP than the group B with a median OS of 13.0 months. Among 82 patients enrolled in the study, AFP responders (*n* = 22) exhibited increased median OS and PFS compared with nonresponders (*n* = 13) (13.0 versus 9.0 months and 8.0 versus 4.0 months, respectively). We observed lower median OS and PFS for AFP responders in comparison with the previous studies. This discrepancy may be attributed to the following reasons. (1) The proportion of pretreatment AFP levels above 400 ng/ml was higher in both groups. (2) There are many prognostic factors in HCC patients with PVTT. (3) The sample size in our study was smaller.

Wada et al. [21] analyzed 62 advanced HCC patients with extrahepatic spread (EHS) or MVI, in which 15 were treated with the combination therapy of sorafenib and three-dimensional conformal radiotherapy (3DCRT) using a total irradiation dose of 30–60 Gy, while 47 underwent only sorafenib treatment. The median PFS of MVI or EHS, PFS of whole lesions, and overall survival in the sorafenib plus 3DCRT group were longer than the sorafenib group (13.5, 10.6, and 31.2 versus 3.3, 3.5, and 12.1 months, respectively). Que et al. [22] evaluated the efficacy of SBRT with and/or without sorafenib for advanced HCC with PVTT and found that SBRT combined with sorafenib improved response rate (77.77% versus 75.00% for SBRT only), median PFS (6.0 versus 3.0 months for SBRT only), median OS (12.5 versus 7.0 months for SBRT only), and OS, albeit the trends did not reach statistical significance. In the current study, we showed similar results with the previous studies. Compared with group B (IMRT alone), group A (IMRT plus sorafenib) displayed a favorable trend in median OS (11.0 versus 9.0 months, *P*=0.036), 1 yr and 2 yrs OS (44.9% versus 28.6% and 3.8% versus 2.6%), median PFS (6.0 versus 3.0 months, *P*=0.012), 1 yr and 2 yrs PFS (20.7% versus 2.7% and 6.9% versus 0.0%). Though differences in overall response between the two groups did not achieve statistical significance (*P* > 0.05), the ORR of group A was better than group B, and patients in group A had lower disease progression than those in group B. Meanwhile, we found that some patients in group A had access to other advanced and effective treatment during follow-up. Thus, the present study further confirms the importance and effectiveness of combination therapy.

Recently, Chen et al. [23] analyzed sixty-one HCC patients with tumor thrombosis in the main portal vein or vena cava to assess the response and survival of radiotherapy. In the study, the tumor thrombosis exhibited a higher response rate than the primary tumor (83.7% versus 77.1%) in the field of radiotherapy, while the primary failure pattern was the outfield progression. Kim et al. [24] reported that pre-SBRT multisegment recurrence was significantly correlated with poor outfield intrahepatic control, and failures in the liver outside the PTV remained a problem. Similarly, the outfield intrahepatic recurrence was the main failure pattern for patients treated with IMRT and sorafenib in the present study. Notably, patients undergoing IMRT combined with sorafenib displayed favorable DMFS (10.0 versus 8.0 months), primary tumor response rate (61.1% versus 45.7%), and tumor thrombosis response rate (61.1% versus 54.4%) compared with those who did not. Therefore, we reason that the combination of local and systemic treatment may help to reduce the outfield recurrence. On the contrary, the main failure pattern for patients treated with IMRT alone was in-field recurrence, implying that the recurrence may be related to the inadequate radiation dose. Thus, it remains necessary to determine the optimal dose of radiation and combination therapy for reducing the recurrence rate.

Prognosis in HCC patients with PVTT varies according to patient's clinical characteristics, tumor biological aggressiveness, degree of portal system invasion, complications caused by portal hypertension, and patient's tolerance to anti-tumor therapy [25]. Choi et al. [26] investigated prognostic significance of PVTT response in 100 HCC patients treated with localized concurrent chemoradiotherapy. Among all the patients, those with a response both in tumor and in PVTT had the most prolonged median OS (16.7 versus 8.4 months for patients with response neither in tumor nor in PVTT). Moreover, multivariate analysis revealed that both ORR of the tumor and CR of the PVTT were independent prognostic factors for OS. Kong et al. [27] identified gender, biologically effective dose (BED), ECOG, Child–Pugh classification and types of tumor thrombi as prognostic factors for OS and found that high-BED level group (≥58.9 Gy) achieved a better median OS than the low-BED level group (42.0 versus 19.0 months, *P*=0.016). In the current study, a Cox proportional hazards regression model showed a reduced risk of death in patients with Child–Pugh class A, AFP reduction, and primary tumor remission. Thus, our findings may contribute to more effective treatment decisions based on risk stratification.

Acute toxicity induced by radiotherapy combined with sorafenib treatment remains an issue that should not be neglected. Chen et al. [28] conducted a phase 2 study on combined sorafenib and RT for 40 patients with unresectable HCC, in which 24 with PVTT were treated with RT with concurrent and sequential sorafenib. In the study, they observed that during RT, the incidences of hand-foot skin reactions grade ≥2, diarrhea, and hepatic toxicities grade ≥2 were 37.5%, 25.0%, and 35.0%, respectively. After RT, nine patients experienced grade 3 or higher hepatic toxicities; among them, six developed RILD. Based on these observations, they proposed that the combination should be used with caution. Goody et al. [29] analyzed 43 HCC patients with unresectable liver metastases to determine maximum tolerated dose (MTD) and toxicities of sorafenib combined with SBRT or whole liver radiotherapy (WLRT). In the study, one or more grade 3+ acute toxicity was observed in eleven (33.0%) patients, at a median time of 10 days starting from radiotherapy. Moreover, sorafenib and 21.6 Gy in 6 fraction WLRT resulted in two deaths from nonclassic liver toxicity. Thus, they reasoned that the combination of WLRT and sorafenib contributed to the high rate of liver toxicity. In our study, although the incidence of grade 1-2 nausea (41.7%), anorexia (61.1%), abdominal pain (55.6%), fatigue (75.0%), and skin reactions (55.6%) in the IMRT with sorafenib group were higher than those in the IMRT only. The hematologic toxicity mainly included leukopenia and thrombocytopenia. The grade 3 leukopenia and thrombocytopenia were present in 8.3% and 13.9% of patients receiving IMRT with sorafenib. Five patients including 2 (5.6%) in the IMRT with sorafenib group showed grade 3 increased level of GGT, while one patient died from deterioration of liver function, ascites, and icterus 10 months after the end of treatment, which was associated with tumor progression. Although there was no RILD in either group, the incidence of hepatic toxicities should not be ignored. Given that most HCC patients are associated with liver cirrhosis, the combination treatment requires the strict assessment of liver function and tolerance in patients.

There are still some limitations in the present study. Firstly, this study was retrospective, and the treatment pattern before radiotherapy was not wholly consistent. Secondly, the total sample size was relatively small. Thirdly, the nonrandomized design in this study may lead to a selection bias even though there was no difference in the primary clinical data between the two groups. The large prospective studies are required for validating the efficacy and safety of IMRT concurrent with sorafenib.

## 5. Conclusion

Despite the availability of new targeted drugs, sorafenib is still preferred for PVTT targeted treatment of PVTT patients due to economic and national insurance reasons. Radiotherapy can be a good choice to further improve the efficacy on the basis of the original treatment. In this study, compared with patients undergoing IMRT only, those treated with IMRT and sorafenib concurrently displayed favorable median OS, PFS, and DMFS, while achieving relatively high level of treatment response and reduced levels of AFP with an acceptable level of toxicity. Therefore, IMRT combined with sorafenib could serve as an alternative therapeutic strategy in clinical practice.

## Figures and Tables

**Table 1:** Clinical characteristics of patients.

Variable	IMRT + sorafenib (*n* = 36)	IMRT (*n* = 46)	*P* value
	*n* (%)	*n* (%)	
Gender			0.362
Male	31 (86.1)	36 (78.3)	
Female	5 (13.9)	10 (21.7)	
Age			0.328
≤55	13 (36.1)	12 (26.1)	
>55	23 (63.9)	34 (73.9)	
Tumor size (cm)			0.459
≤5	14 (38.9)	23 (50.0)	
>5–≤10	16 (44.4)	15 (32.6)	
>10	6 (16.7)	8 (17.4)	
AJCC TNM stage			0.236
III	18 (50.0)	29 (63.0)	
IV	18 (50.0)	17 (37.0)	
ECOG			1.000
≤1	32 (88.9)	40 (87.0)	
>1	4 (11.1)	6 (13.0)	
Etiology			0.588
HBV	30 (83.3)	36 (78.3)	
HCV	2 (5.6)	4 (8.7)	
None HBV, none HCV	4 (11.1)	6 (13.0)	
Antiviral therapy			0.153
Yes	18 (50.0)	25 (54.3)	
No	18 (50.0)	21 (45.7)	
Child–Pugh classification			1.000
A	35 (97.2)	44 (95.7)	
B	1 (2.8)	2 (4.3)	
ALT levels (IU/L)			0.854
≤40	30 (83.3)	39 (84.8)	
>40–≤100	5 (13.9)	6 (13.0)	
>100	1 (2.8)	1 (2.2)	
AFP levels (*μ*g/L)			0.390
≤20	7 (19.4)	15 (32.6)	
20–400	10 (27.8)	9 (19.6)	
≥400	19 (52.8)	22 (47.8)	
PVTT typing			0.544
II	12 (33.3)	13 (28.3)	
III	15 (41.7)	29 (63.0)	
IV	9 (25.0)	4 (8.7)	
Radiation dose (Gy)			0.120
≤50	19 (52.8)	32 (69.6)	
>50	17 (47.2)	14 (30.4)	

IMRT, intensity-modulated radiotherapy; AJCC, American Joint Committee on Cancer; ECOG, eastern cooperative oncology group; HBV, hepatitis B virus; HCV, hepatitis C virus; ALT, alanine aminotransferase; AFP, alpha-fetoprotein; PVTT, portal vein tumor thrombosis.

**Table 2:** Overall response, AFP response, and failure pattern after treatment.

	IMRT + sorafenib (*n* = 36)	IMRT (*n* = 46)	*P* value
	*n* (%)	*n* (%)	
Overall response			0.120
CR	0 (0.0)	1 (2.2)	
PR	22 (61.1)	20 (43.5)	
SD	10 (27.8)	13 (28.3)	
PD	4 (11.1)	12 (26.0)	
AFP response			0.003
≥20%	22 (61.1)	13 (28.3)	
<20%	14 (38.9)	33 (71.7)	
Failure pattern			
In-field recurrence	7 (19.4)	16 (34.8)	0.125
Out-field recurrence	14 (38.9)	12 (26.1)	0.216
Distant metastasis	6 (16.7)	8 (17.4)	0.931

CR, complete response; PR, partial response; SD, stable disease; PD, progression disease.

**Table 3:** Univariate and multivariate analysis of OS in the enrolled cohort.

Variable	No. of cases	Univariate analysis		Multivariate analysis	
		HR (95% CI)	*P* value	HR (95% CI)	*P* value
Gender (male versus female)	67/15	0.53 (0.29–0.96)	0.036		
Age (≤50 versus >50)	25/57	0.89 (0.52–1.53)	0.669		
Tumor size (cm) (≤5/>5–≤10/>10)	37/31/14		0.852		
≤5 versus >10	37/14	1.00 (0.51–1.96)	0.991		
>5–≤10 versus >10	31/14	0.87 (0.43–1.74)	0.688		
AJCC TNM stage (III versus IV)	47/35	1.00 (0.62–1.61)	0.986		
ECOG (≤1 versus >1)	72/10	1.44 (0.68–3.04)	0.338		
Etiology (HBV/HCV/none HBV, none HCV)	66/6/10		0.849		
HBV versus none HBV, none HCV	66/10	0.83 (0.41–1.68)	0.597		
HCV versus none HBV, none HCV	6/10	0.76 (0.23–2.48)	0.647		
Antiviral therapy (yes versus no)	43/39	1.47 (0.89–2.42)	0.131		
Child–Pugh classification (A versus B)	79/3	0.14 (0.03–0.67)	0.014	0.12 (0.03–0.60)	0.010
ALT levels, IU/L (≤40/>40–≤100/>100)	69/11/2		0.152		
≤40 versus >100	69/2	0.89 (0.12–6.49)	0.909		
> 40–≤100 versus >100	11/2	1.75 (0.22–13.82)	0.594		
AFP levels (*μ*g/L) (≤20/20–400/≥400)	22/19/41		0.035		
≤20 versus ≥400	22/41	1.95 (1.10–3.47)	0.022		
20–400 versus ≥400	19/41	0.89 (0.47–1.66)	0.704		
PVTT typing (II/III/IV)	22/44/13		0.878		
II versus IV	22/13	1.09 (0.53–2.27)	0.811		
III versus IV	44/13	1.18 (0.61–2.28)	0.622		
Radiation dose (Gy) (≤50 versus >50)	51/31	1.69 (1.02–2.81)	0.042		
Overall response (response versus no response)	43/39	0.41 (0.25–0.69)	0.001	0.41 (0.24–0.68)	0.001
AFP response (≥20% versus <20%)	35/47	0.55 (0.33–0.91)	0.019	0.51 (0.30–0.84)	0.009
Sorafenib (yes versus no)	36/46	0.55 (0.33–0.90)	0.018		

**Table 4:** Acute toxicity.

Adverse event	IMRT + sorafenib (*n* = 36)			IMRT (*n* = 46)			*P* value
	Grade 1 (%)	Grade 2 (%)	Grade 3 (%)	Grade 1 (%)	Grade 2 (%)	Grade 3 (%)	Any grade
Nausea	11 (30.6)	4 (11.1)	0 (0.0)	12 (26.1)	1 (2.2)	0 (0.0)	0.146
Vomiting	6 (6.7)	2 (5.6)	0 (0.0)	8 (17.4)	0 (0.0)	0 (0.0)	0.515
Anorexia	19 (52.8)	3 (8.3)	0 (0.0)	22 (47.8)	1 (2.2)	0 (0.0)	0.223
Abdominal pain	18 (50.0)	2 (5.6)	0 (0.0)	16 (34.8)	1 (2.2)	0 (0.0)	0.085
Fatigue	24 (66.7)	3 (8.3)	0 (0.0)	22 (47.8)	2 (4.3)	0 (0.0)	0.035
Skin reactions	18 (50.0)	2 (5.6)	0 (0.0)	16 (34.8)	0 (0.0)	0 (0.0)	0.043
Leukopenia	12 (33.3)	12 (33.3)	3 (8.3)	23 (50.0)	7 (15.2)	1 (2.2)	0.056
Thrombocytopenia	12 (33.3)	11 (30.6)	5 (13.9)	11 (23.9)	13 (28.3)	6 (13.0)	0.452
Anemia	4 (11.1)	5 (13.9)	1 (2.8)	10 (21.7)	3 (6.5)	0 (0.0)	0.799
Increased ALT level	12 (33.3)	3 (8.3)	0 (0.0)	12 (26.1)	1 (2.2)	0 (0.0)	0.166
Increased AST level	19 (52.8)	1 (2.8)	0 (0.0)	18 (39.1)	1 (2.2)	0 (0.0)	0.209
Increased GGT level	18 (50.0)	7 (19.4)	2 (5.6)	17 (37.0)	7 (15.2)	3 (6.5)	0.239

## Data Availability

The data supporting the results in the manuscript can be obtained from the corresponding author Yao Tan based on reasonable requests.

## References

[1] Bray F., Ferlay J., Soerjomataram I., Siegel R. L., Torre L. A., Jemal A. (2018). Global cancer statistics 2018: GLOBOCAN estimates of incidence and mortality worldwide for 36 cancers in 185 countries. *CA: A Cancer Journal for Clinicians*.

[2] Chen W., Sun K., Zheng R. (2018). Cancer incidence and mortality in China, 2014. *Chinese Journal of Cancer Research*.

[3] Zhang Z.-m., Lai E. C. H., Zhang C. (2015). The strategies for treating primary hepatocellular carcinoma with portal vein tumor thrombus. *International Journal of Surgery*.

[4] Wu Y. L., Feng Q. F. (2019). Progress of surgery combined with radiotherapy for primary hepatocellular carcinoma. *Chinese Journal of Radiation Medicine and Protection*.

[5] Bruix J., Raoul J. L., Sherman M. (2012). Efficacy and safety of sorafenib in patients with advanced hepatocellular carcinoma: subanalyses of a phase III trial. *Journal of Hepatology*.

[6] Kudo M., Finn R. S., Qin S. (2018). Lenvatinib versus sorafenib in first-line treatment of patients with unresectable hepatocellular carcinoma: a randomised phase 3 non-inferiority trial. *Lancet (London, England)*.

[7] Zhu K., Chen J., Lai L. (2014). Hepatocellular carcinoma with portal vein tumor thrombus: treatment with transarterial chemoembolization combined with sorafenib—a retrospective controlled study. *Radiology*.

[8] Giorgio A., Merola M. G., Montesarchio L. (2016). Sorafenib combined with radio-frequency ablation compared with sorafenib alone in treatment of hepatocellular carcinoma invading portal vein: a western randomized controlled trial. *Anticancer Research*.

[9] Brade A. M., Ng S., Brierley J. (2016). Phase 1 trial of sorafenib and stereotactic body radiation therapy for hepatocellular carcinoma. *International Journal of Radiation Oncology, Biology, Physics*.

[10] Zhao Y., Zhu X., Wang H. (2019). Safety and efficacy of transcatheter arterial chemoembolization plus radiotherapy combined with sorafenib in hepatocellular carcinoma showing macrovascular invasion. *Frontiers in Oncology*.

[11] Shi J., Lai E. C., Li N. (2011). A new classification for hepatocellular carcinoma with portal vein tumor thrombus. *Journal of Hepato-Biliary-Pancreatic Sciences*.

[12] Eisenhauer E. A., Therasse P., Bogaerts J. (2009). New response evaluation criteria in solid tumours: revised RECIST guideline (version 1.1). *European Journal of Cancer (Oxford, England: 1990)*.

[13] Gomaa A. I., Hashim M. S., Waked I. (2014). Comparing staging systems for predicting prognosis and survival in patients with hepatocellular carcinoma in Egypt. *PLoS One*.

[14] European Association for the Study of the Liver (2018). EASL clinical practice guidelines: management of hepatocellular carcinoma. *Journal of Hepatology*.

[15] Kok V. C., Chen Y. C., Chen Y. Y. (2019). Sorafenib with transarterial chemoembolization achieves improved survival vs. sorafenib alone in advanced hepatocellular carcinoma: a nationwide population-based cohort study. *Cancers*.

[16] Yoon S. M., Ryoo B. Y., Lee S. J. (2018). Efficacy and safety of transarterial chemoembolization plus external beam radiotherapy vs sorafenib in hepatocellular carcinoma with macroscopic vascular invasion: a randomized clinical trial. *JAMA Oncology*.

[17] He M., Li Q., Zou R. (2019). Sorafenib plus hepatic arterial infusion of oxaliplatin, fluorouracil, and leucovorin vs sorafenib alone for hepatocellular carcinoma with portal vein invasion: a randomized clinical trial. *JAMA Oncology*.

[18] He C., Peng W., Liu X., Li C., Li X., Wen T. F. (2019). Post-treatment alpha-fetoprotein response predicts prognosis of patients with hepatocellular carcinoma: a meta-analysis. *Medicine*.

[19] Chou W. C., Lee C. L., Yang T. S. (2018). Changes in serum alpha-fetoprotein level predicts treatment response and survival in hepatocellular carcinoma patients and literature review. *Journal of the Formosan Medical Association*.

[20] Sánchez A. I. P., Roces L. V., García I. Z. (2018). Value of alpha-fetoprotein as an early biomarker for treatment response to sorafenib therapy in advanced hepatocellular carcinoma. *Oncology Letters*.

[21] Wada Y., Takami Y., Matsushima H. (2018). The safety and efficacy of combination therapy of sorafenib and radiotherapy for advanced hepatocellular carcinoma: a retrospective study. *Internal Medicine (Tokyo, Japan)*.

[22] Que J., Wu H. C., Lin C. H., Huang C. I., Li L. C., Ho C. H. (2020). Comparison of stereotactic body radiation therapy with and without sorafenib as treatment for hepatocellular carcinoma with portal vein tumor thrombosis. *Medicine*.

[23] Chen B., Li Y. X., Wang W., Tan Y., Song Y. W. (2019). Efficacy and prognosis of radiotherapy for hepatocellular carcinoma with tumor thrombosis in main portal vein or/and vena cava. *International Journal of Radiation Oncology, Biology, Physics*.

[24] Kim J. W., Kim D. Y., Han K. H., Seong J. (2019). Phase I/II trial of helical IMRT-based stereotactic body radiotherapy for hepatocellular carcinoma. *Digestive and Liver Disease: Official Journal of the Italian Society of Gastroenterology and the Italian Association for the Study of the Liver*.

[25] Cerrito L., Annicchiarico B. E., Iezzi R., Gasbarrini A., Pompili M., Ponziani F. R. (2019). Treatment of hepatocellular carcinoma in patients with portal vein tumor thrombosis: beyond the known frontiers. *World Journal of Gastroenterology*.

[26] Choi Y., Kim J. W., Cha H., Han K. H., Seong J. (2014). Overall response of both intrahepatic tumor and portal vein tumor thrombosis is a good prognostic factor for hepatocellular carcinoma patients receiving concurrent chemoradiotherapy. *Journal of Radiation Research*.

[27] Kong X. Q., Dong Y. P., Wu J. X. (2017). High-biologically effective dose palliative radiotherapy for a tumor thrombus might improve the long-term prognosis of hepatocellular carcinoma: a retrospective study. *Radiation Oncology (London, England)*.

[28] Chen S. W., Lin L. C., Kuo Y. C., Liang J. A., Kuo C. C., Chiou J. F. (2014). Phase 2 study of combined sorafenib and radiation therapy in patients with advanced hepatocellular carcinoma. *International Journal of Radiation Oncology, Biology, Physics*.

[29] Goody R. B., Brade A. M., Wang L. (2017). Phase I trial of radiation therapy and sorafenib in unresectable liver metastases. *Radiotherapy and Oncology: Journal of the European Society for Therapeutic Radiology and Oncology*.

